# Proteasome Function Is Required for Biological Timing throughout the Twenty-Four Hour Cycle

**DOI:** 10.1016/j.cub.2011.03.060

**Published:** 2011-05-24

**Authors:** Gerben van Ooijen, Laura E. Dixon, Carl Troein, Andrew J. Millar

**Affiliations:** 1 School of Biological Sciences and Centre for Systems Biology at Edinburgh, University of Edinburgh, The King's Buildings, Mayfield Road, Edinburgh EH9 3JD, UK

## Abstract

Circadian clocks were, until recently, seen as a consequence of rhythmic transcription of clock components, directed by transcriptional/translational feedback loops (TTFLs). Oscillations of protein modification were then discovered in cyanobacteria [1, 2]. Canonical posttranslational signaling processes have known importance for clocks across taxa [3–11]. More recently, evidence from the unicellular eukaryote *Ostreococcus tauri* revealed a transcription-independent, rhythmic protein modification [12] shared in anucleate human cells [13]. In this study, the *Ostreococcus* system reveals a central role for targeted protein degradation in the mechanism of circadian timing. The *Ostreococcus* clockwork contains a TTFL involving the morning-expressed CCA1 and evening-expressed TOC1 proteins [14]. Cellular CCA1 and TOC1 protein content and degradation rates are analyzed qualitatively and quantitatively using luciferase reporter fusion proteins. CCA1 protein degradation rates, measured in high time resolution, feature a sharp clock-regulated peak under constant conditions. TOC1 degradation peaks in response to darkness. Targeted protein degradation, unlike transcription and translation, is shown to be essential to sustain TTFL rhythmicity throughout the circadian cycle. Although proteasomal degradation is not necessary for sustained posttranslational oscillations in transcriptionally inactive cells, TTFL and posttranslational oscillators are normally coupled, and proteasome function is crucial to sustain both.

## Highlights

► CCA1 protein degradation rate is clock regulated ► Sensitivity of the circadian clock to proteasomal inhibition is phase independent ► Nontranscriptional rhythms only rely on the proteasome while coupled to the TTFL

## Results and Discussion

### CCA1 Degradation Is Clock Regulated, and TOC1 Degradation Is Dark Responsive

**Figure 1:**
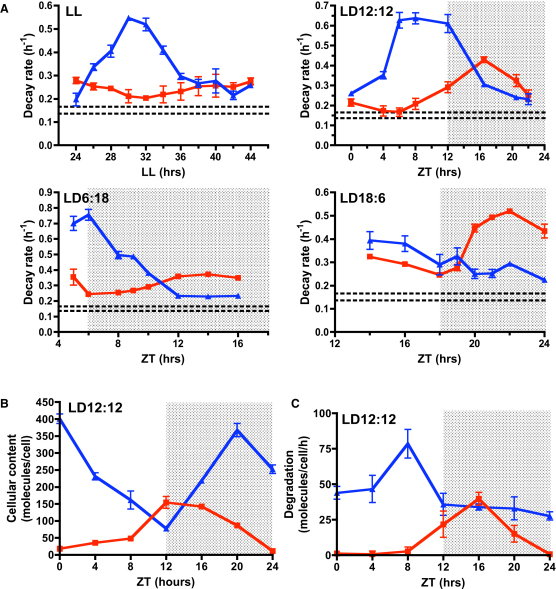
CCA1-LUC and TOC1-LUC Degradation Rates under Different Light Regimes (A) Degradation rates of CCA1-LUC (blue traces) and TOC1-LUC (red traces) calculated from the curve fitting to the exponential phase of decay following inhibition of de novo protein synthesis with cycloheximide. The x axis indicates treatment time; light regime is indicated in the panels. Error bars represent standard error of the mean (SEM; n = 5). Decay rates measured for free luciferase ranged from 0.165 to 0.136 hr^−1^, as indicated by the horizontal dotted lines. (B) Number of CCA1-LUC (blue line) or TOC1-LUC (red line) molecules/cell for an LD12:12 cycle calculated by in vitro luciferase activity of cell extracts (mean values plotted ± SEM; n = 2). (C) Absolute degradation rates in molecules/cell/hr for CCA1-LUC (blue lines) and TOC1-LUC (red lines) obtained from multiplying decay rates by molecule number (mean values plotted ± SEM; n = 2). See also Figure S1.

The *Ostreococcus* transcription factor CIRCADIAN CLOCK ASSOCIATED-1 (CCA1) and response regulator TIMING OF CAB1 EXPRESSION (TOC1) have recently been shown to function similarly to the *Arabidopsis thaliana* orthologs, forming a transcriptional/translational feedback loop (TTFL) thought to be central to the circadian clock mechanism [14, 15]. *Ostreococcus* lines expressing CCA1 or TOC1 from their native promoters as translational fusions to firefly luciferase were previously characterized [14]. pCCA1::CCA1-LUC and pTOC::TOC1-LUC lines will be referred to as CCA1-LUC and TOC1-LUC. To comprehensively analyze the degradation rates of CCA1-LUC and TOC1-LUC throughout the circadian cycle, we blocked de novo protein synthesis using saturating concentrations [12] of cycloheximide (CHX) at 2 hr intervals in constant light (LL). Decay rates were calculated from curve fitting to the initial exponential decay of the CCA1-LUC or TOC1-LUC trace following treatment (the data and fitted decay rates are shown in Figures S1A–S1D available online). CCA1 degradation rates showed a peak in the middle of the subjective day (30 hr into LL, or 6 hr after anticipated dawn; Figure 1A), roughly coinciding with the trough in CCA1 protein expression under light:dark (LD) cycles (Figures S1A–S1D). The diurnal peak was at ∼0.6 hr^−1^, 2- or 3-fold higher than the trough rate in the subjective night. This result revealed rhythmic CCA1 protein degradation in constant conditions.

The TOC1 degradation rate, in contrast, varied little in LL (0.2–0.27 hr^−1^), prompting us to test its regulation under physiologically relevant diurnal cycles. Assays in cultures under cycles of 12 hr light:12 hr dark (LD12:12) showed that the TOC1-LUC degradation rate was higher in darkness (Figure 1A). Because elements of LD regulation of TOC1 degradation were previously reported [16, 17], we tested TOC1 degradation rates around the transition to darkness under long (LD18:6) or short (LD6:18) days. A sharp increase in TOC1 degradation was evident in long-day conditions but less clear in short-day conditions until later at night, suggesting that some circadian gating exists on the increased TOC1 degradation in response to darkness (Figure 1A). Peak TOC1 decay rates were always higher (up to 2-fold) in darkness compared to LL, although the peak time varied depending on day length.

The CCA1-LUC decay rate in LD12:12 peaked from Zeitgeber Time 6 (ZT6), as in LL, although the peak was significantly broader (Figure 1A). In LD6:18, the CCA1-LUC degradation rate again peaked at ZT6 but fell rapidly in darkness to a low level by ZT12, similar to its profile in LL. We conclude that the degradation profile of CCA1-LUC is circadian controlled and additionally shaped by the light:dark cycle, possibly because of the higher levels of CCA1 observed under long days compared to short days [18].

### Quantitative Analysis of Cellular Clock Protein Content and Degradation Rate

Decay rates measured as above will reflect the actual protein degradation plus the deactivation rate of the luciferase enzyme [19], assuming that these very different processes are independent. The decay rate for luciferase, dominated by the deactivation rate [19], was measured by two approaches. First, cultures containing the transcriptional reporter fusions pCCA1::LUC and pTOC1::LUC were treated with CHX (Figure S1E), revealing decay rates slower (between 0.153 and 0.165 hr^−1^) than rates found with the translational fusions. Second, the rhythmic activity profile of free luciferase in pTOC1::LUC cultures was monitored in short-day LD cycles, because TOC1 promoter activity is close to zero in darkness. Luciferase decay rates over several nights were observed between 0.136 and 0.143 hr^−1^ (Figure S1F). The observed rates were close to the value (0.18 hr^−1^) previously estimated by fitting a mathematical model of the *Ostreococcus* clock to a large set of luciferase data [18]. In conclusion, deactivation of luciferase constitutes only a minor fraction of the peak rate of rhythmic CCA1 degradation but a substantial fraction of the lowest rate, as indicated by the horizontal dotted lines in Figure 1A.

Using an in vitro luciferase activity assay, the number of CCA1-LUC or TOC1-LUC molecules per cell was estimated under LD12:12 by comparing the activity of cell extracts from a known number of cells to a recombinant luciferase standard of known activity. CCA1-LUC cycled between peak and trough levels of close to 400 and 80 molecules/cell, and TOC1-LUC cycled between 150 and 10 molecules/cell (Figure 1B). Subsequently, multiplying decay rates (hr^−1^) with molecule counts (molecules/cell) would allow an estimation of absolute degradation at any time in molecules/cell/hr. To negate the effect of luciferase deactivation, we subtracted the average rate value (0.15 hr^−1^) from the decay rates. Absolute CCA1-LUC degradation (Figure 1C) peaked around ZT8, relating to ∼75 molecules/cell/hr. Higher degradation rates of CCA1 thus contributed to reaching the trough level of CCA1 protein around ZT9 (Figure S1B). The increased level of TOC1-LUC decay in darkness related to ∼40 molecules/hr.

The low molecule numbers in the crowded cellular environment could indicate that stochastic effects might not be trivial in the *Ostreococcus* clock. Comparisons between deterministic and stochastic models of the *Ostreococcus* clock using a range of molecule numbers, including the levels experimentally observed here, have previously revealed that stochasticity indeed has potentially significant effects on free-running behavior and desynchronization of cells [20].

These results, utilizing the unique advantages *Ostreococcus tauri* offers, expand previous knowledge from *Arabidopsis* and lead to the first estimates of clock component molecule number per cell in any plant or algal model organism, as well as, to our knowledge, the most detailed in vivo analysis of rhythmic clock component degradation rates.

### *Ostreococcus* Shares Components of *Arabidopsis* Clock Protein Degradation Pathways

*Arabidopsis* CCA1 homolog LATE ELONGATED HYPOCOTYL (LHY) is rapidly degraded in vitro by the proteasome in plant extracts [21] in a process negatively regulated by DE-ETIOLATED1 (DET1). DET1 has also been shown to regulate the degradation of transcription factors involved in light signaling, together with ubiquitin ligase CONSTITUTIVE PHOTOMORPHOGENIC 1 (COP1) [22]. The *Ostreococcus* genome encodes an ortholog of the *Arabidopsis* DET1 and COP1 proteins (CAL55339 and CAL53135, respectively). *Arabidopsis* TOC1 is targeted for degradation via the F-box protein ZEITLUPE (ZTL) in a dark-dependent fashion [16]. Because the *Ostreococcus* genome contains a homolog of ZTL (CAL53380), this protein might contribute to TOC1 degradation, although the algal protein lacks the chromophore-binding LOV domain of ZTL. Other factors that regulate TOC1 stability in *Arabidopsis* (PRR3 [23], FKF1 and LKP2 [24], and GI [25]) are not conserved.

*Ostreococcus* DET1, COP1, and ZTL were recently found to be differentially expressed under LD12:12 cycles. ZTL peaked in the night, and DET1 peaked during the day (Figure S1G, adapted from [26]), consistent with roles in degrading TOC1 at night (ZTL) or stabilizing CCA1 during the day (DET1). Further studies will be necessary to fully establish the contributions of protagonists and antagonists of clock component protein stability and their mechanisms of light dependence.

### Effects of Pharmacological Inhibition of Targeted Protein Degradation on Free-Running Behavior

**Figure 2:**
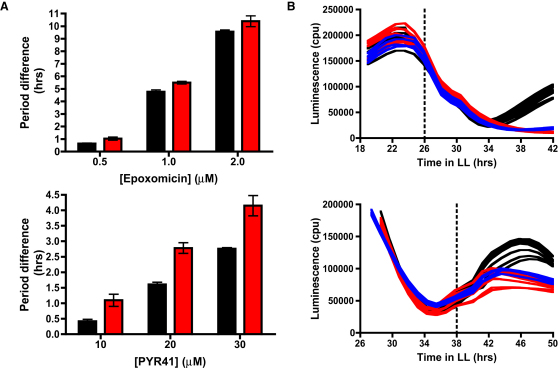
Effects of Proteasomal Inhibition (A) Period difference relative to vehicle-treated cells, resulting from treatment with indicated concentrations of epoxomicin or PYR-41 on CCA1-LUC (red bars) or pCCA1::LUC (black bars). Error bars represent standard deviation (SD; n = 8). (B) Effect of epoxomicin (blue traces, n = 4) or MG132 (red traces, n = 8) on CCA1-LUC in downward (top) or upward (bottom) phase, compared to vehicle (black traces, n = 8). See also Figure S2.

The widely used proteasome inhibitor MG132 increases the free-running period in a dose-dependent fashion in *Ostreococcus* [12]. MG132 is a trileucine aldehyde, inhibiting proteasome subunits β1 and β5, but it also targets papain-like cysteine proteases in plants [27]. To verify that modulating the proteasome pathway indeed affects the *Ostreococcus* free-running period, we analyzed the effects of the highly selective proteasome inhibitor epoxomicin [28], which inhibits all three catalytic proteasome subunits in plants [27]. Low micromolar concentrations of this drug resulted in robust period increases of ∼9–10 hr (Figure 2A and Figure S2A). Furthermore, an inhibitor (PYR-41) [29] that acts on the ubiquitin/proteasome pathway via inhibition of ubiquitin-activating enzymes yielded similar, albeit less potent, effects in the lengthening period (Figure 2A and Figure S2B). In combination, the effects of these inhibitors show that targeted protein degradation via the proteasome is indeed necessary to maintain rhythmicity.

However, when proteasomal inhibitors were applied to CCA1-LUC *Ostreococcus* cells before the peak in CCA1 degradation rate (Figure 1A), no immediate effect was observed on CCA1-LUC traces (Figure 2B, top), and the CCA1 level dropped to trough levels, as in vehicle-treated cells. However, rhythmicity was not sustained because CCA1 levels did not rise at the appropriate phase, possibly meaning that increased CCA1 protein degradation toward trough levels is not directly proteasome mediated, but proteasomal activity is necessary to allow CCA1 levels to rise from trough levels. We consider the most parsimonious explanation to be that proteasomal degradation of a negative regulator is associated with rising CCA1 levels. In line with this hypothesis, when proteasome inhibition was applied at a phase when CCA1-LUC levels were rising (Figure 2B, bottom), the upward trend was curtailed.

Several transcriptional repressors of *CCA1* have been identified in the *Arabidopsis* clock, including members of the pseudo-response regulator (PRR) family [30] to which TOC1 belongs, or the TCP transcription factor CCA1 HIKING EXPEDITION (CHE) [31]. *Ostreococcus* does not contain any TCP transcription factors, and *TOC1* is a single gene that is thought to function as an activator of algal *CCA1* rather than a repressor [18]. It is possible that our results will not be explained by transcriptional control but rather by modulation of negative regulators such as DET1 and COP1 acting on the CCA1 protein itself, as DET1 does on *Arabidopsis* LHY [21].

### Proteasome Inhibition Arrests the Clock Regardless of Phase

Until recently, circadian rhythms were regarded to be dictated by rhythmic expression of core clock proteins: in green cells, mainly LHY/CCA1 and TOC1 [32]. This dogma was challenged in cyanobacteria by the notion that the three clock proteins KaiA, KaiB, and KaiC generated an ∼24 hr rhythmic output in vitro [1, 2], but such biochemical oscillations were not identified in eukaryotes. Underexposed intellectual precedent exists that questions this TTFL model, showing that at least in certain taxa, transcription is not essential for rhythmicity. It was shown that the nucleus was not necessary for rhythmicity in *Acetabularia* [33, 34] and that translational rather than transcriptional control is critical to rhythmicity in *Lingulodinium* (previously known as *Gonyaulax*) [35]. Furthermore, clock networks generally keep oscillating even if core TTFL components are misexpressed [36]. Recent work on *Ostreococcus* has shown that substantial parts of the circadian cycle are insensitive to inhibition of transcription or translation, in contrast to the behavior expected if the TTFL was the only driver of rhythmicity [18], and that posttranslational oscillations persist after several days without TTFL rhythmicity [12].

The observation that degradation rates and molecule numbers of clock proteins are never at 0 (Figure 1) suggested that targeted degradation might set their levels at all phases. An implication would be that proteasome inhibition would potentially alter circadian timing at any phase. The reversible characteristics of proteasome inhibitor MG132 allowed the testing of this hypothesis using pulsed treatments, ended by wash off.

**Figure 3:**
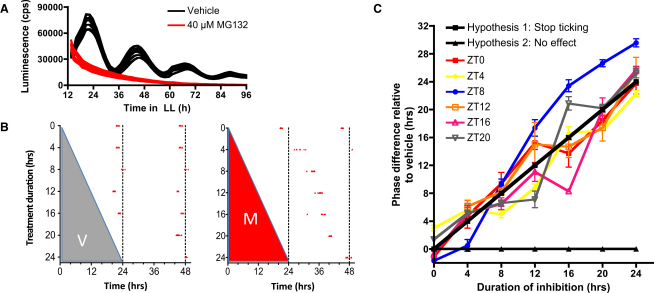
Proteasomal Inhibition Stops TTFL Rhythmicity Phase-Independently (A) Application of saturating concentrations of MG132 (40 μM, red traces) or vehicle (black traces) to CCA1-LUC cells in constant light. (B) Examples from wedge data of peak phases of individual wells (n ≥ 6) of CCA1-LUC cells subjected to various (0–24) hours of proteasomal inhibition (red wedge, right) or vehicle (gray wedge, left) starting at ZT0 and ending by wash off. (C) Summary of phase shifts (error bars represent SD, n ≥ 6) relative to vehicle-treated controls for all treatment durations (x axis) and starting times. Two black lines represent the expected result, assuming either total resetting by wash off (hypothesis 1) or no effect (hypothesis 2). See also Figure S3.

Saturating concentrations of MG132 arrested rhythmic behavior of the CCA1-LUC line (Figure 3A). CCA1-LUC cells entrained in LD12:12 cycles were transferred to constant light at dawn (ZT0), and application of MG132 stopped normal oscillatory behavior. After wash off, the cells directly resumed oscillations (Figure S3A), suggesting that treatment was reversible and largely nontoxic. The delay in phase resulting from treatment pulses followed a direct relation with the duration of the treatment (Figure S3B), suggesting that the circadian pacemaker had paused. The period of the reinitiated rhythm after wash off was not substantially affected (Figure S3C), showing that wash off was efficient and that drug concentration was reduced to insignificant levels. The ability of treated cells to re-entrain was demonstrated by reinstating LD12:12 cycles after 48 hr of constant light (Figure S3A). Altogether, this indicates that when drugs are applied at ZT0, the *Ostreococus* clock is paused by MG132, and cells reset to wash off.

Warranted by the results described above, we exploited pulsed inhibition to comprehensively test sensitivity to proteasomal inhibition throughout a 24 hr cycle. LD12:12-entrained CCA1-LUC cells were subjected to pulses of increasing duration (4, 8, 12, 16, 20, and 24 hr), starting at 4 hr intervals throughout the circadian cycle in constant light. Phase information after wash off was recorded to analyze whether phase systematically departed from vehicle-treated cells. This type of experiment is referred to as a “wedge” experiment [37, 38], named after the anticipated shape of phase outputs, assuming that treatment stops the clock altogether and that cells reset to wash off. If inhibition of a drug target would not affect the clock, the resulting phase should be similar to vehicle-treated cells (Figure 3C). In *Ostreococcus*, inhibition of transcription or translation leads to complex, phase-dependent responses following neither of the two hypotheses [12]. In contrast, pulses of MG132 resulted in exactly the anticipated wedge shape predicted by full resetting to wash off, regardless of what time treatment is started (Figures 3B and 3C and Figure S3G). This result shows that, in contrast to transcription and translation [12], proteasome function determines timekeeping at all phases.

### Rhythmic Sulphonylation of Peroxiredoxin Proteins Is Arrested by Proteasomal Inhibition

**Figure 4:**
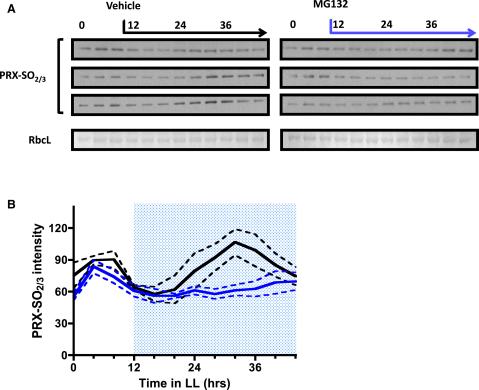
Application of MG132 Arrests Cytosolic Oscillations (A) Three independent 48 hr time series of protein extracts in constant light with vehicle or MG132 treatment starting after 12 hr. Samples were run on immunoblots using a PRX-SO_2/3_ antibody. For equal loading control, Coomassie staining of Rubisco (RbcL) is shown on a representative gel (bottom panels). (B) Densitometry performed with ImageJ64 showing grouped data of the three replicates for vehicle-treated (black line) or MG132-treated (blue line) cells. Dotted lines indicate SD; blue shaded area indicates window of inhibition. See also Figure S4.

Peroxiredoxins are an evolutionarily conserved group of antioxidant enzymes. Scavenging reactive oxygen species drives hyperoxidation of a redox-active cysteine to sulphonic acid (sulphonylation), which in turn drives formation of homo-oligomers [39, 40]. Circadian sulphonylation of peroxiredoxin proteins (PRX) was observed in transcriptionally incompetent *Ostreococcus* cells [12], as well as in mature (naturally anucleate) human red blood cells [13], indicating that TTFLs alone cannot explain all circadian outputs. Unlike most basic physiological processes in this photosynthetic organism [26, 41], rhythmic peroxiredoxin modification persisted in darkness [12]. Blocking transcription, translation, or light fails to stop this rhythm, so we investigated whether inhibition of targeted protein degradation would do so in conditions in which the other three factors were uncompromised. LD12:12-entrained cells were transferred to constant light, and rhythmic PRX sulphonylation was analyzed on western blots using an antibody specifically targeting the sulphonylated PRX-SO_2/3_ forms. Vehicle-treated cells showed strong rhythms in PRX modification in LL (Figure 4). When MG132 was applied 12 hr into constant light, PRX-SO_2/3_ rhythmicity was paused or dramatically slowed (Figure 4). Thus, proteasome function is necessary for posttranslational rhythmicity in cells that are competent to synthesize new proteins. This result indicates that the nontranscriptional oscillator is strongly coupled to the TTFL, such that an arrhythmic TTFL can stop or severely perturb the nontranscriptional rhythm.

We next explored the effect of proteasome inhibition on the nontranscriptional oscillator in cells that were incompetent for de novo TTFL component synthesis, i.e., *Ostreococcus* cells in constant darkness. Pharmacological treatments acting on posttranslational modulators had similar effects on the period of PRX rhythmicity in these cells as they did on TTFL rhythms in cells under constant light [12], showing that multiple biochemical processes are involved in generating the nontranscriptional rhythm. We hypothesized that proteasome function would not be among them (so MG132 would not affect PRX rhythms in constant darkness), because if posttranslational rhythms relied on targeted protein degradation, they could not persist over several days without de novo synthesis. Indeed, rhythmic sulphonylation of PRX was still observed with saturating concentrations of MG132 in darkness (Figure S4).

These results indicate, more clearly than experiments using transgenic lines with altered period length [12], that the TTFL and posttranslational oscillators are strongly coupled under physiologically relevant conditions and that proteasome function is crucial to sustain their joint operation. However, posttranslational oscillations are uncoupled from proteasomal degradation in darkness, when the TTFL appears to be absent rather than inhibited. It is unclear which of many cellular changes between light-grown and darkened algal cells allow this uncoupling of the posttranslational oscillator. Similar behavior was observed in mammalian cells [13], comparing anucleate red blood cells (TTFL absent, PRX rhythmic) and *cry1cry2* mutant fibroblasts (TTFL inhibited, PRX arrhythmic or aberrant).

Shutdown of cellular transcription/translation and an apparent independence from proteasomal degradation might reflect a survival mechanism for *Ostreococcus* cells in nature when oceanic currents carry them to places where light levels are insufficient to provide energy for functions such as cell division and protein synthesis. The fact that cells in this near-dormant state are still oscillating suggests that rhythmicity should be seen as a fundamental property of living cells that exists even in cells that gain no apparent benefit from anticipating the solar cycle, like human red blood cells [13].

Based on recent identification of posttranslational components in clocks across taxa [1, 2, 4, 12, 13, 42], this work explores the contributions of rhythmic proteolysis in eukaryotic cellular timekeeping. A free-running peak in CCA1 degradation rate is reported, whereas TOC1 degradation is accelerated in darkness. Furthermore, proteasomal degradation of a negative regulator might be involved in CCA1 synthesis. Proteasomal inhibition is shown to stop transcriptional clock output at any phase. Taking into account that transcription/translation is not required throughout the full circadian cycle to sustain rhythmicity in *Ostreococcus*, this study establishes the position of rhythmic targeted protein degradation as not only a central element but also a constant requirement for timekeeping, at least in this organism.

## Experimental Procedures

### Culturing

Materials were ordered from Sigma-Aldrich (UK) unless indicated. *Ostreococcus tauri* cells were grown under 12:12 hr blue (Ocean Blue, Lee lighting filter 724) LD cycles (17.5 μE/m^2^) in artificial sea water (Instant Ocean) supplemented with Keller marine enrichment nutrients, hereafter referred to as ASW.

### Imaging

Bioluminescent imaging was performed on a TopCount (Packard) fitted with red and blue LED lights (5–12 μE/m^2^ depending on position in plates) in white 96-well plates (Lumitrac, Greiner Bio-one). Five to seven days before recording, cells were plated at a density of 5–10 × 10^6^ cells/ml and kept under entrainment conditions. One day before imaging, 150 μl ASW was replaced with 150 μl ASW containing 333 μM luciferin. Period and phase analyses were carried out using the fast Fourier transform (FFT)-nonlinear least squares or mFourfit functions, respectively, in BRASS 3 software [43], based on data spanning at least three circadian cycles (with the exception of period and phase estimates in Figures S3B, S3C, S3E, and S3F, where just two cycles of LL data were used). Outputs were manually confirmed.

### Pharmacology

Drugs were dissolved in dimethyl sulfoxide, diluted in ASW, and added to the saturated or otherwise indicated concentrations. Saturating concentrations are: CHX, 1 μg/ml; MG132, 40 μM; epoxomicin, 10 μM. For data in Figure 1 and Figures S1A–S1D, n = 5, vehicle control n = 3. For Figure 2B, n = 8 for MG132 and n = 4 for epoxomicin. For Figure 2A, Figure 3, and Figure S3, treatment and vehicle controls are n = 8.

### Calculating Degradation Rates

Luminescence measurements in the exponential decay phase following CHX treatment (0.5 to 3.7 hr after treatment) were used to calculate degradation rates, as detailed in the legend to Figure S1.

### Luciferase Assays

Analysis of CCA1 and TOC1 molecule counts (Figure 1B) was performed by comparing luciferase activity in cell extracts prepared from a known number of cells (as analyzed in a haemocytometer) in 5 ml culture with a commercial luciferase standard of known concentration in the Luciferase Reporter Gene Detection Kit (Sigma-Aldrich, LUC-1).

### Wedge Experiment

Wedge experiments were performed as detailed in [12] and in the legend to Figure S3.

### Western Blotting

Western blot analyses of PRX modification were performed as described in [12] and in the legend to Figure S4. Densitometry was performed using ImageJ64 (W. Rasband, National Institute of Mental Health).

## Supplemental Information

Document S1. Four Figures
