# Supplementary material for: Proteasome Function Is Required for Biological Timing throughout the Twenty-Four Hour Cycle
Source: Curr Biol. 2011 May 24;21(10):869–75. doi: 10.1016/j.cub.2011.03.060 (PMC3102177; doi:10.1016/j.cub.2011.03.060)
Supplement: Document S1. Four Figures [file mmc1.pdf]

Current Biology, Volume *21*

Supplemental Information

Proteasome Function Is Required for Biological Timing throughout the Twenty-Four Hour Cycle

Gerben van Ooijen, Laura E. Dixon, Carl Troein, and Andrew J. Millar

A

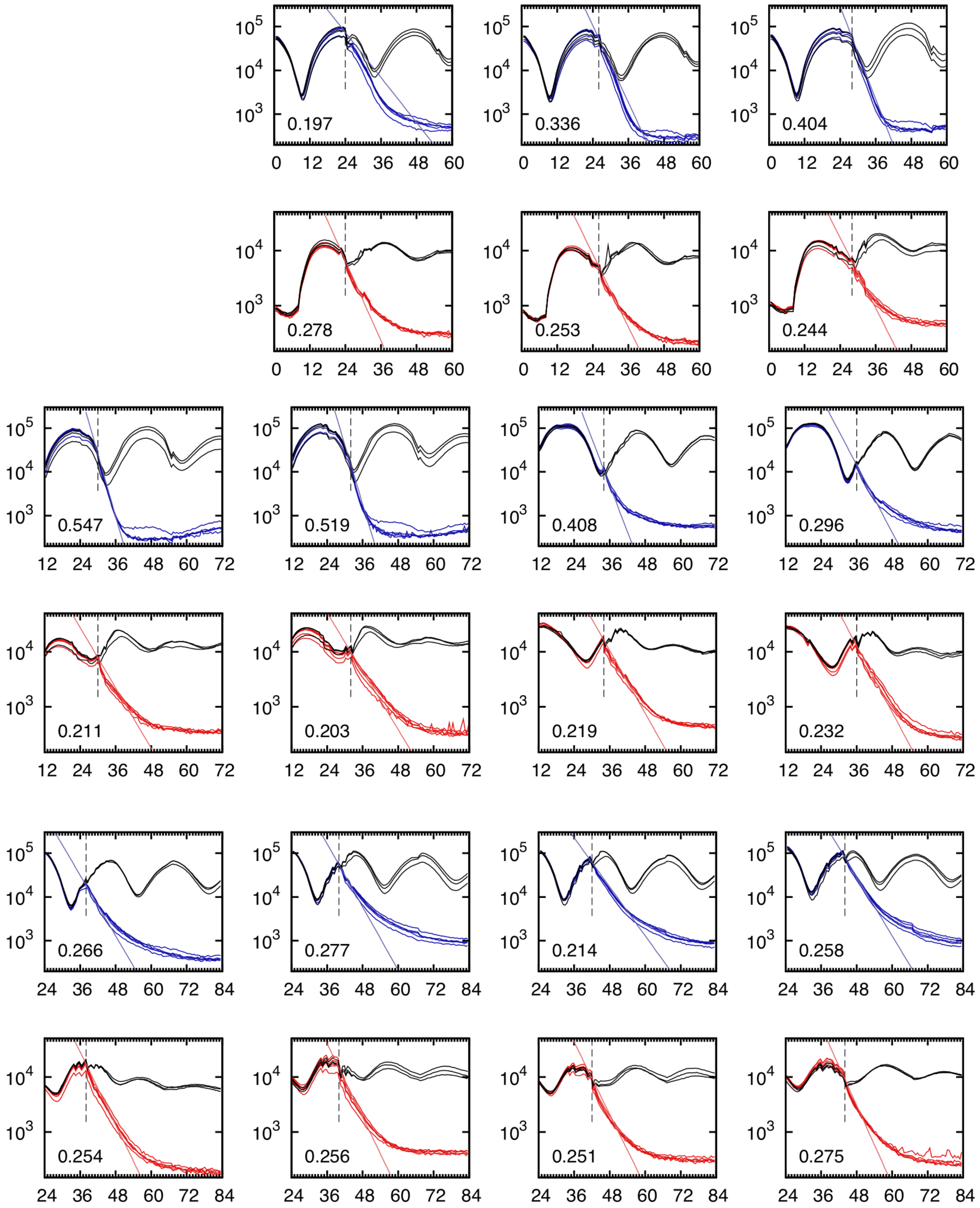
0.197
0.336
0.404
0.278
0.253
0.244
0.547
0.519
0.408
0.296
0.211
0.203
0.219
0.232
0.266
0.277
0.214
0.258
0.254
0.256
0.251
0.275

B

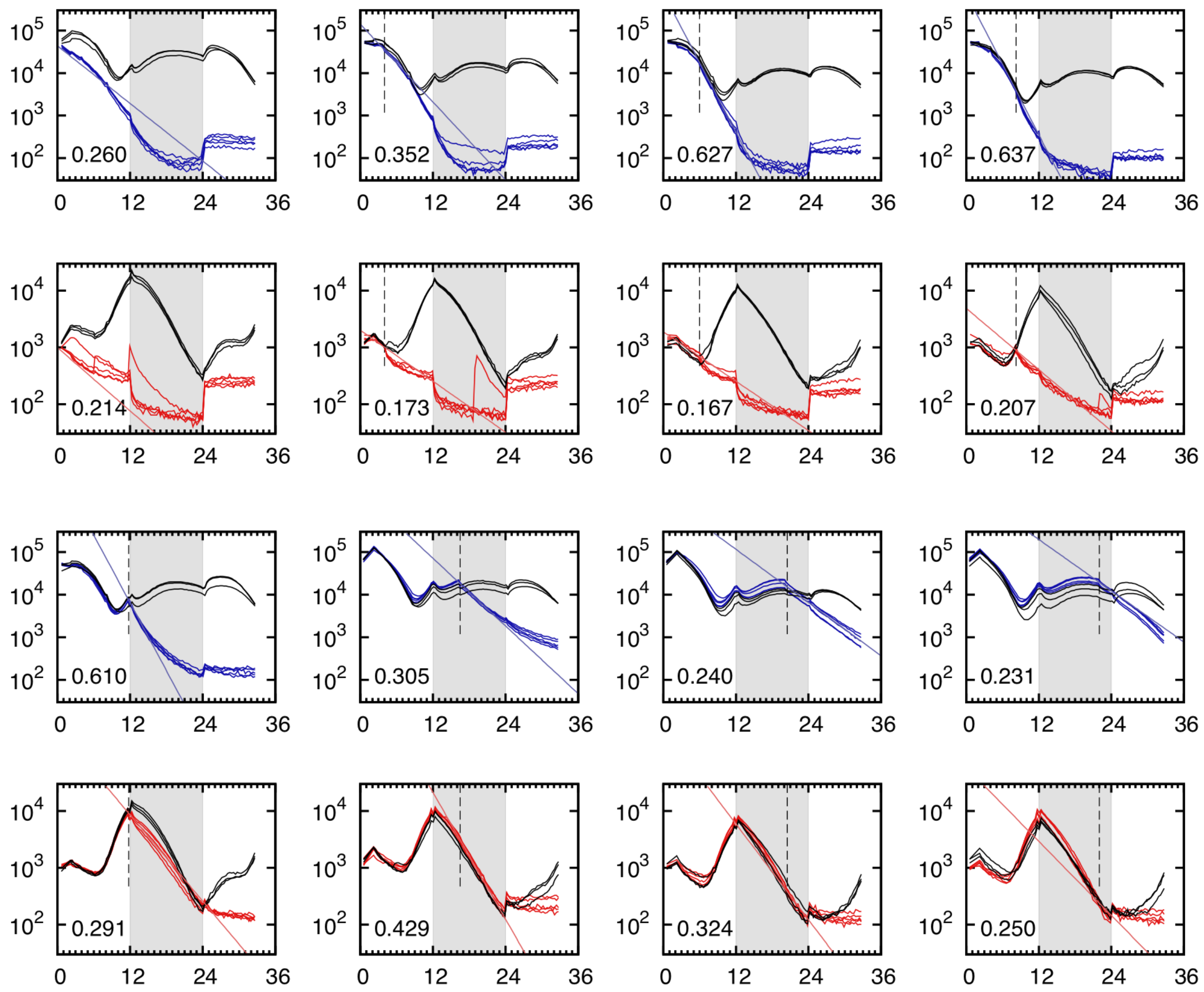
0.260
0.352
0.627
0.637
0.214
0.173
0.167
0.207
0.610
0.305
0.240
0.231
0.291
0.429
0.324
0.250

C

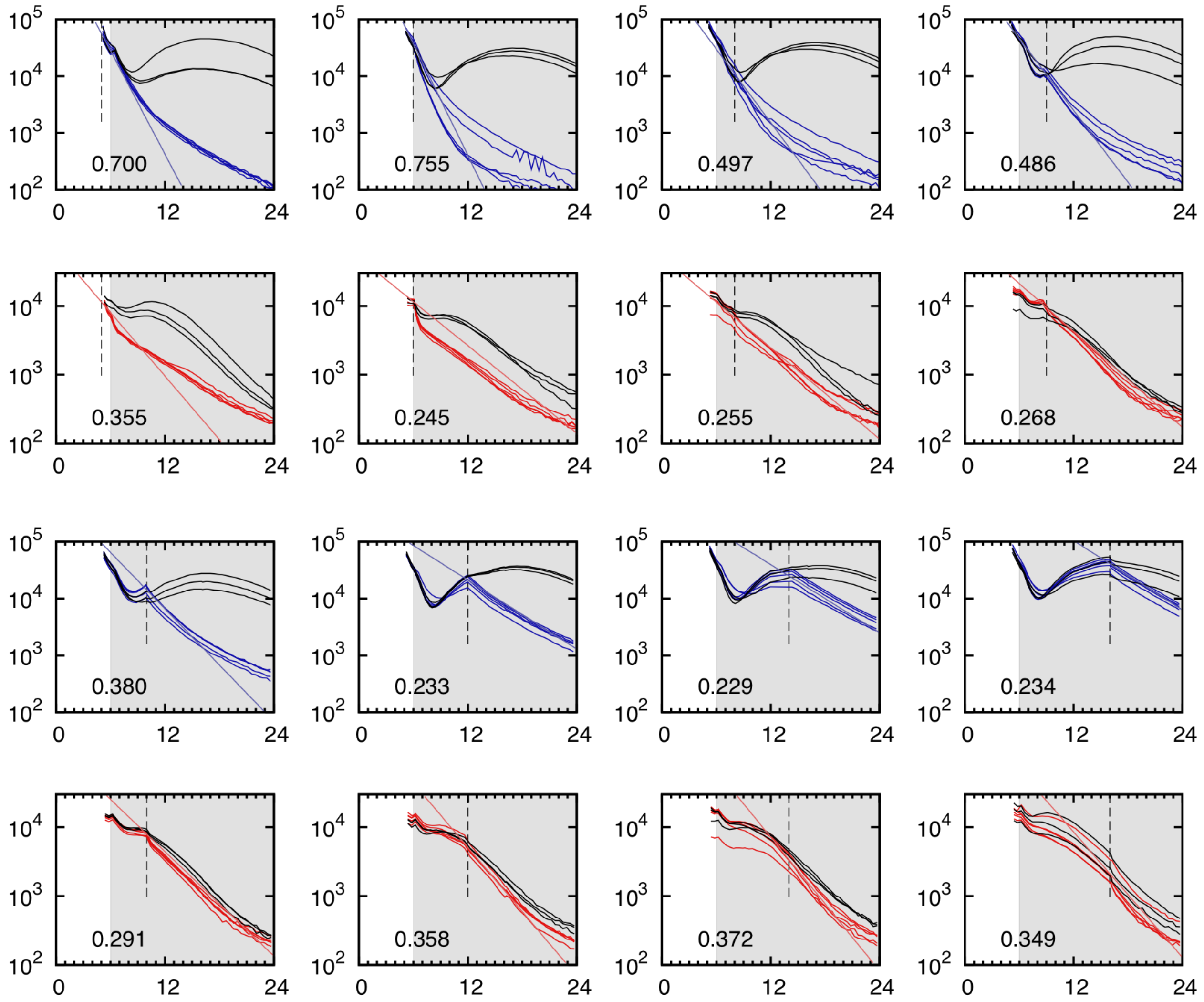
0.700
0.755
0.497
0.486
0.355
0.245
0.255
0.268
0.380
0.233
0.229
0.234
0.291
0.358
0.372
0.349
10^5
10^4
10^3
10^2
0
12
24

D

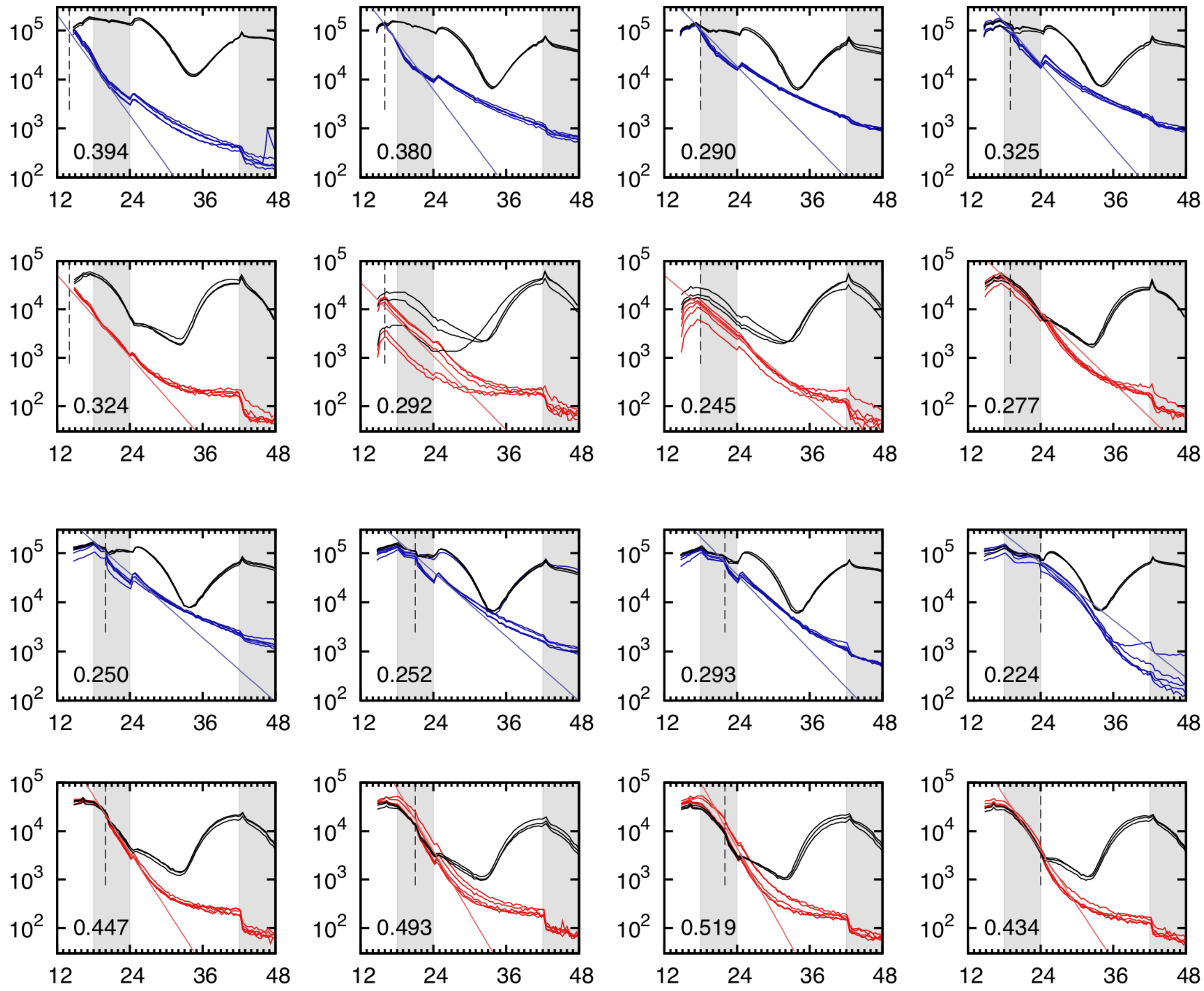
0.394
0.380
0.290
0.325
0.324
0.292
0.245
0.277
0.250
0.252
0.293
0.224
0.447
0.493
0.519
0.434
12
24
36
48

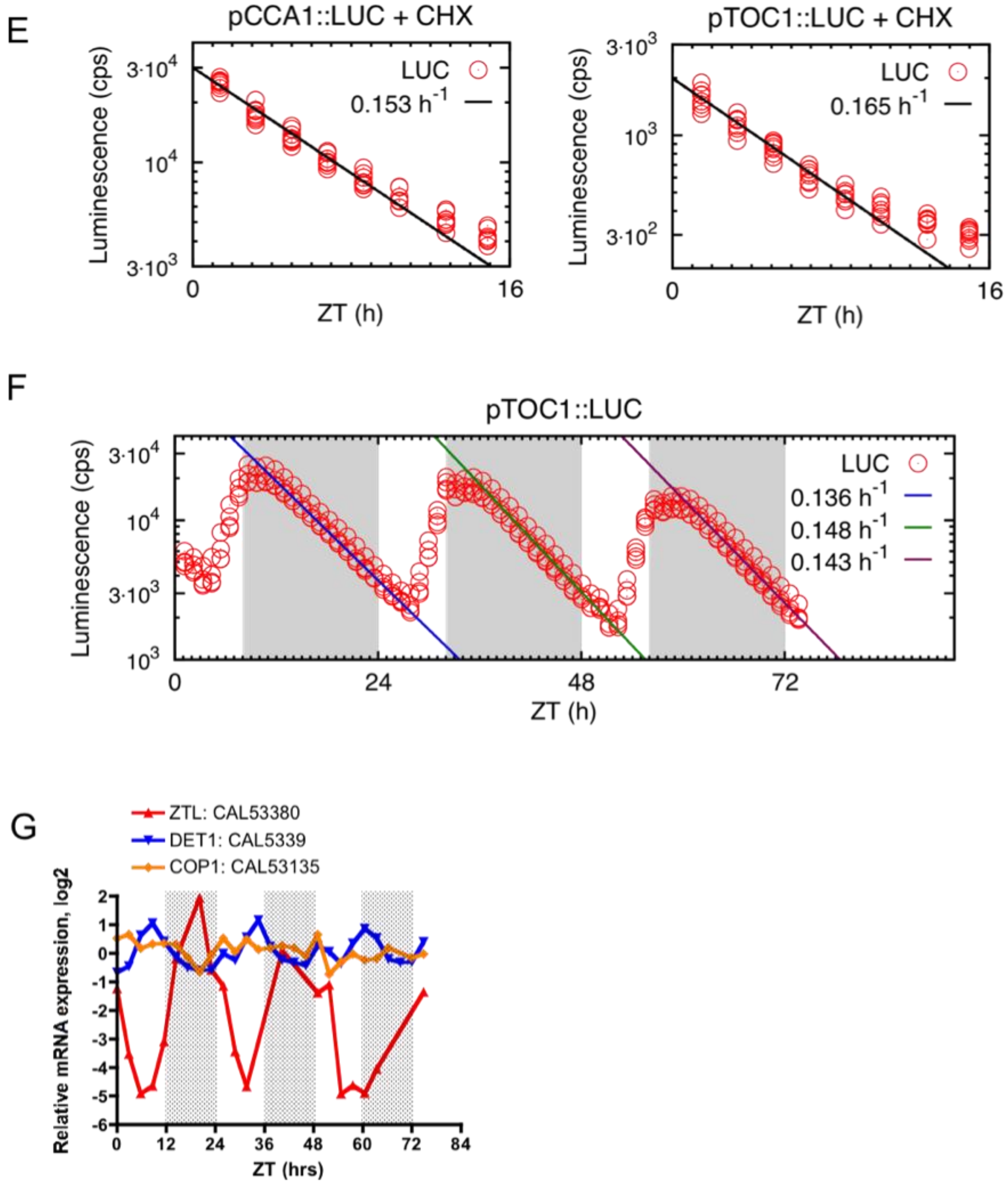
E
pCCA1::LUC + CHX
pTOC1::LUC + CHX
Luminescence (cps)
LUC
0.153 h-1
0.165 h-1
ZT (h)
F
pTOC1::LUC
LUC
0.136 h-1
0.148 h-1
0.143 h-1
G
ZTL: CAL53380
DET1: CAL5339
COP1: CAL53135
Relative mRNA expression, log2
ZT (hrs)

Figure S1. Data Used for Calculating Clock Protein Decay Rates, Related to Figure 1

(A-D) Luminescent traces on a log scale, from all experimentation used to compute degradation rates plotted in Figure 1A. De novo protein synthesis was inhibited with CHX (CCA1-LUC; blue lines, TOC1-LUC; red lines, n=5) compared to vehicle (black lines, n=3). This experiment was performed on the second day of constant light (A), or in LD12:12 (B), LD6:18 (C), or LD18:6 (D). Straight lines on this logarithmic scale represent fits to the data, and the decay rate d (h^{-1}) is indicated. Dotted lines indicate treatment time. For each pair of consecutive time points, the decay rates of the five replicate wells were computed, and reduced to their mean and standard deviation. Subsequently, the mean of the estimated decay rates was computed across the pairs of consecutive time points, weighted by the reciprocal of the standard deviation. This procedure was found to be more robust than alternative methods.

(E) The luciferase deactivation rate was estimated by linear regression of the exponential signal decay of luciferase expressed from the CCA1 (pCCA1::LUC) or TOC1 (pTOC1::LUC) promoter, following treatment with CHX ($n = 4$, $SD = 0.005\ h^{-1}$ for both lines).

(F) As an independent means, luciferase decay was measured over several nights using pTOC1::LUC in short day conditions ($n = 8$, $SD \leq 0.003\ h^{-1}$, using ZT 14-24 of each day).

(G) Rhythmic transcription in Ostreococcus cells in LD12:12 cycles of proteins involved in TOC1 or CCA1 stability in Arabidopsis (data adapted from [26]).

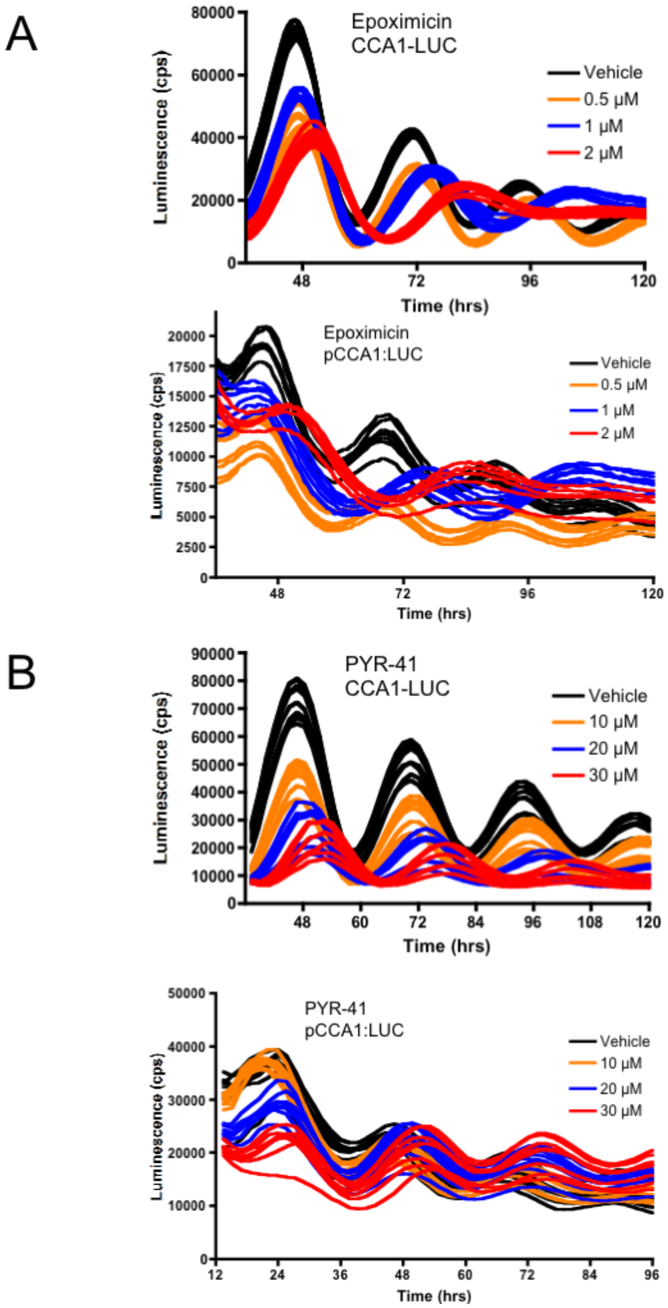

Figure S2. Representative Free-Running Traces in Response to Pharmacological Inhibition, Related to Figure 2

Traces of CCA1-LUC (top panels) and pCCA1-LUC (lower panels) resulting from inhibition with indicated concentrations of inhibitors against the proteasome (epoxomicin, A) or Ubiquitin ligases (PYR-41, B).

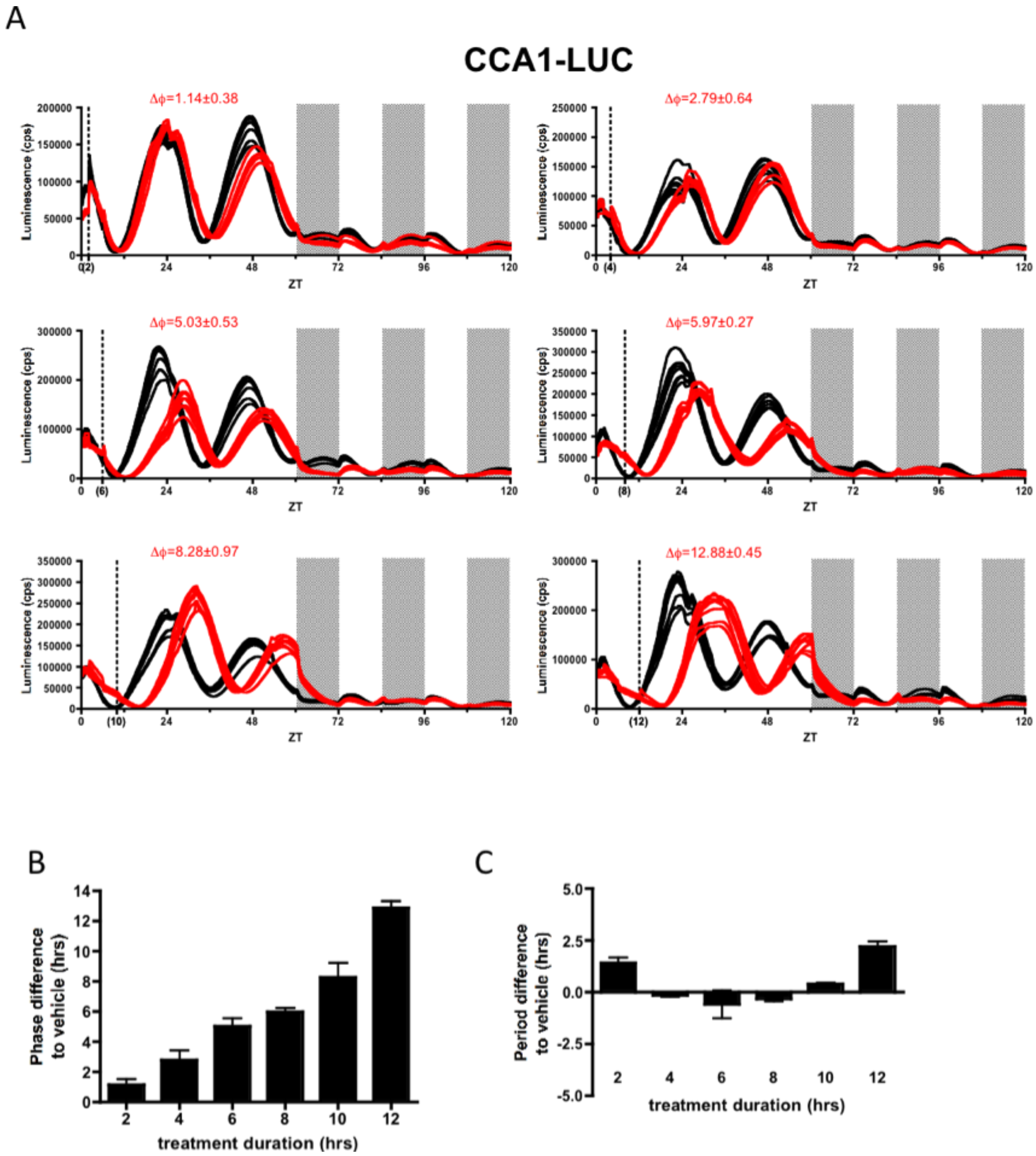

A
CCA1-LUC
Δϕ=1.14±0.38
Δϕ=2.79±0.64
Δϕ=5.03±0.53
Δϕ=5.97±0.27
Δϕ=8.28±0.97
Δϕ=12.88±0.45
Luminescence (cps)
ZT
B
Phase difference to vehicle (hrs)
treatment duration (hrs)
C
Period difference to vehicle (hrs)
treatment duration (hrs)

D

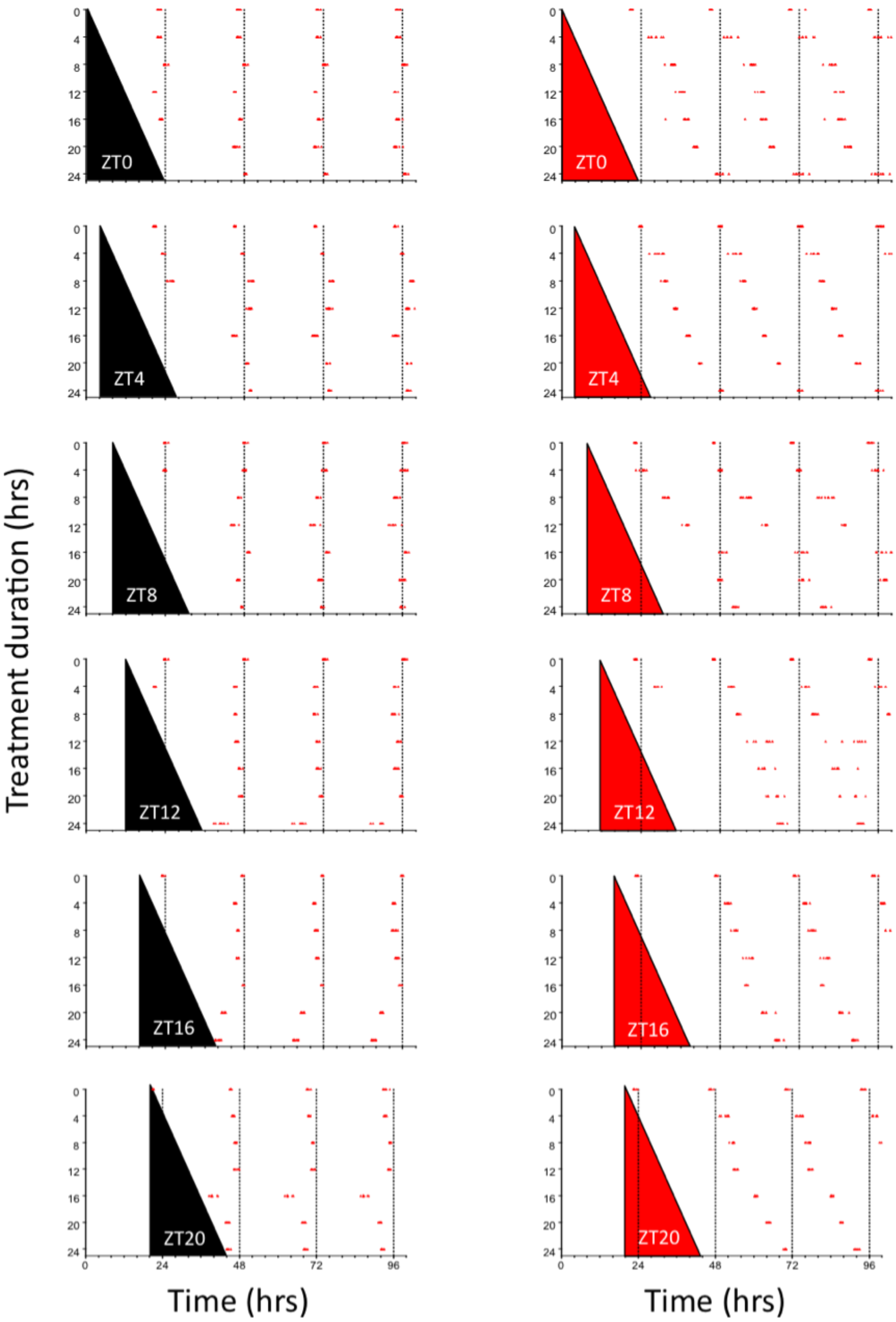
Treatment duration (hrs)
ZT0
ZT4
ZT8
ZT12
ZT16
ZT20
ZT0
ZT4
ZT8
ZT12
ZT16
ZT20
0
24
48
72
96
Time (hrs)
Time (hrs)

Figure S3. Full Results of Pulsed Treatments with Proteasome Inhibition, Related to Figure 3

For wash-off of MG132 (A-D), all ASW was pipetted from the cell aggregates that naturally form in the bottom of microplate wells, and quickly but gently washed with fresh ASW plus luciferin, using an 8-channel pipette. The effects of epoxomicin were found to be irreversible ([27] and data not shown). However, the highly comparable qualitative and quantitative effects of MG132 and epoxomicin (Figure 2) strongly indicated that inhibitory effects on the proteasome at least strongly dominate the effects observed using MG132.

(A) Traces from CCA1-LUC lines entrained in 12:12 LD cycles and released into LL for 2 days at ZT0, with MG132 (red traces) or vehicle treatment (black traces) starting at transfer to LL and for increasing duration. Wash-off is indicated by black dotted lines. Phase (ϕ) change (± SD, n=8) resulting from treatment relative to vehicle treatment is indicated. After 2 days, a 12:12 light/dark regime was reinstated to check for the cells' ability to re-entrain to a photoperiod (indicated with black shades).

(B-C) Effects of treatment depicted in A) on phase (B) and period (C) of subsequent CCA1-LUC oscillations.

(D) Full results of wedge experiment with proteasome inhibition. Peak times of individual replicates of CCA1-LUC expression in constant light after entrainment in 12:12 LD cycles and treatment with vehicle (left hand graphs, black wedges) or MG132 (right hand graphs, red wedges) starting from ZT0, 4, 8, 12, 16, or 20 extending for 4-hour increments up to 24 hours ($n \geq 6$).

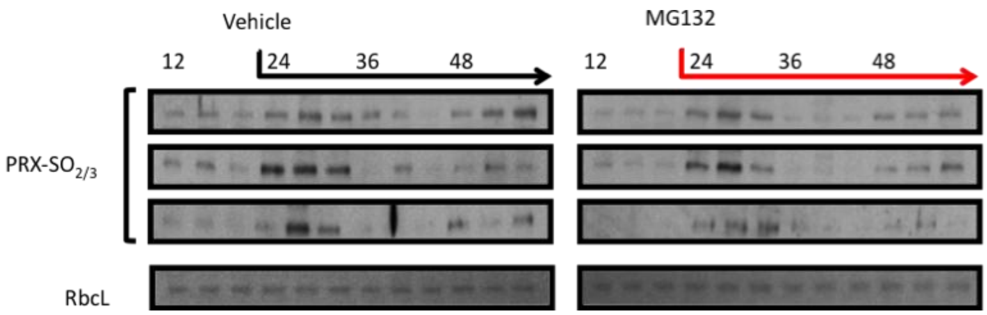

Figure S4. Application of MG132 Does Not Arrest Cytosolic Oscillations in Transcriptionally Inactive Cells, Related to Figure 4
Three independent 48-hour time series of protein extracts starting 12 hours into DD (ZT0), with vehicle or MG132 applied 24 hours into DD (ZT12). For equal loading control, coomassie staining of Rubisco (RbcL) is shown on a representative gel (lower panels). 5 ml of cells were quickly chilled on ice and centrifuged for 10 minutes at 4 °C at 4,000 g, supernatant was discarded and cells were resuspended in 50 µl ASW, lysed by addition of 50 µl 2x extraction buffer (from LUC-1 detection kit), and mixed with 100 µl 2x Laemmli sample buffer before loading on a 4-12% NuPage Novex Bis-Tris midi gel (Invitrogen). For sampling in constant darkness, ASW was supplemented with 200 mM sorbitol and 0.4% glycerol.

Gels were blotted using an iBlot system (Invitrogen) and following transfer, RbcL protein remaining in the gel was stained as a loading control using SimplyBlue SafeStain (Invitrogen). Immunostaining was performed using a rabbit polyclonal Peroxiredoxin-$SO_{2/3}$ antibody (Abcam ab16830) combined with HRP-labelled secondary antibodies (Abcam ab6721).
